# Editorial: Advances in statistical methods for the genetic dissection of complex traits in plants

**DOI:** 10.3389/fpls.2024.1357564

**Published:** 2024-01-15

**Authors:** Yuan-Ming Zhang, Zhenyu Jia, Shang-Qian Xie, Jia Wen, Shibo Wang, Ya-Wen Zhang

**Affiliations:** ^1^ College of Plant Science and Technology, Huazhong Agricultural University, Wuhan, China; ^2^ Department of Botany and Plant Sciences, University of California, Riverside, Riverside, CA, United States; ^3^ Department of Animal, Veterinary & Food Sciences, University of Idaho, Moscow, ID, United States; ^4^ Department of Genetics, University of North Carolina at Chapel Hill, Chapel Hill, NC, United States; ^5^ International Genome Center, Jiangsu University, Zhenjiang, China

**Keywords:** multi-omics profiling, plants, 3VmrMLM, genome-wide association study, complex traits

## 1 Multi-locus genome-wide association study methods

In real data analysis, most commonly used genome-wide association study (GWAS) methods often miss some important loci and trait heritability. To address these challenges, Li et al. (2022a) established an innovative method named 3VmrMLM based on a compressed variance component mixed model. In 3VmrMLM, all the effects in quantitative trait nucleotide (QTN), QTN-by-environment interaction (QEI), and QTN-by-QTN interaction (QQI) detection are compressed into an effect-related vector, while all polygenic backgrounds are compressed into a vector-related polygenic background. This method is especially well suited for species with a high proportion of heterozygous genotypes, such as human, forests, chrysanthemums, and grasslands.

Can 3VmrMLM replace existing methods? The answer is no, despite 3VmrMLM demonstrating superiority over existing methods. For the detection of loci dominated by additive effects, existing methods remain appropriate, as observed in rice, wheat, and soybean. Since GWAS is based on linkage disequilibrium from historical recombination, there is complementarity between methods (Zhang et al., 2019). However, existing methods face challenges in detecting dominant effects and small allele substitution effects (Zhang et al., 2023).

When analyzing real data, the inflation factor or quantile–quantile plot serves as a common metric to assess method performance. However, this is not crucial for our mrMLM and 3VmrMLM methods (Zhang et al., 2020; Li et al., 2022a), because their genome-wide scanning aims to select potentially associated markers rather than identify significant loci. A method is considered effective when it mines some importantly known and candidate genes around these loci, supported by strong evidence, as seen in 3VmrMLM. These identified loci may be used for genomic selection (Su et al., 2024), while more associated known and candidate genes can be mined and highlighted in the Manhattan plot.

This Research Topic contains three articles focusing on methodological studies and comparisons. Yang et al. proposed the MTOTC method to transform hierarchical data of ordinal traits into continuous phenotypes, which were then analyzed by multi-locus methods. This showed that the combination of MTOTC with any multi-locus method detects more QTNs. To identify QQI via the IIIVmrMLM software (Li et al., 2022b), Han et al. performed Levene’s test to obtain the top 5,000 loci for each trait, and these loci were used to detect QTNs and QQIs associated with 11 flowering time-related traits in 199 Arabidopsis accessions with 216,130 markers. Around 89 QTNs and 130 QQIs, 34 identified genes were reported in previous studies, while 20 candidate genes were predicted; in particular, *AT1G12990* and *AT1G09950* around QQIs may have an interaction effect on flowering time. In addition, He et al. measured five free amino acid levels in 448 rice accessions across two environments, used nine GWAS methods to perform association analysis between phenotypes and 4,325,832 SNPs, and identified 88 stable QTLs, demonstrating the advantages of 3VmrMLM, including the most common QTNs, the highest LOD score, and the highest proportion of all stable QTLs.

## 2 The applications of new multi-locus GWAS methodologies in the genetic dissection of complex traits

Yield is one of the paramount breeding objectives, with nine articles in the Research Topic focusing on identifying QTNs and/or QEIs for yield-related traits. Zhang et al. used 3VmrMLM to re-associate 44,000 SNPs with eight yield-related traits from 413 rice accessions across three environments. They identified 87 known genes around QTNs and QEIs, including *OsMADS5* and *FZP*. Differential expression, functional enrichment, and haplotype analysis revealed the association of *LOC_Os04g53210* and *LOC_Os07g42440* with yield, while *LOC_Os04g53210* around a QEI potentially influenced flowering time. Zhao et al. employed 3VmrMLM to perform association analysis between three measured grain size traits of 159 rice accessions in two environments and 2,017,495 SNPs, identifying 393 QTNs and 8 QEIs. They found 22 genes around QTNs and 2 genes around QEIs to be genuinely associated with these traits. Additionally, 14 candidate genes were significant in differential expression, GO annotation, and haplotype analysis. Moreover, in a joint analysis of main crop and ratoon rice, 4 known genes, 8 additional candidate genes, and 2 candidate gene-by-environment interactions (GEIs) were identified as responsible for grain size-related traits.

Shu et al. evaluated plant height (PH) and ear height (EH) in 203 maize inbred lines at five locations and used 3VmrMLM to perform association analysis between phenotypes and 73,174 SNPs. They detected 23 significant QEIs and 53 corn belt-specific QTNs for the two traits. Transcriptomic and haplotype analysis highlighted the EH-related QEI S10_135 and the PH-related QEI S4_4, as well as corn belt-specific QTNs (S10_4 and S7_1), showcasing the power of 3VmrMLM in QEI discovery. Sun et al. measured the tassel branch number (TBN) of 190 F_2_ individuals and F_2:3_ families, using four methods to associate the phenotypes with 4,136 SNPs. They identified 13 QTLs and 22 QTNs, including large-effect QTLs qTBN6.06-1 and qTBN6.06-2 on chromosome 6. RNA-seq analysis revealed 5 candidate genes associated with TBN. Wen et al. identified 76 QTNs and 73 QEIs for three yield-related traits in 300 tropical and subtropical maize lines with 332,641 SNPs under well-watered, drought, and heat-stress conditions. They reported 34 genes from previous studies, confirming genes associated with drought tolerance (*ereb53* and *thx12*) and heat stress (*hsftf27* and *myb60*). Differential expression, tissue-specific expression, and haplotype analysis confirmed 24 candidate genes, while three yield GEIs (*GRMZM2G064159*, *GRMZM2G146192*, and *GRMZM2G114789*) were predicted.

Feng et al. measured the boll weight (BW) of 290 cotton accessions in nine environments and used GEMMA to perform association analysis between the phenotypes and 25,169 SNPs and 2,315 InDels, identifying two major QTLs on chromosomes A08 and D13. *Ghir_A08G009110* and *Ghir_D13G023010* were confirmed by both transcript-level and differential expression analysis between high- and low-BW accessions during the ovule development stage. Liu et al. measured three seed size-related traits in 196 mung bean accessions across two environments and used four methods to perform association analysis between the phenotypes and 3,607,508 SNPs. *VrKIX8*, *VrPAT14*, *VrEmp24/25*, *VrIAR1*, *VrBEE3*, *VrSUC4*, and *Vrflo2* around QTNs were homologous to known seed development genes in rice and *Arabidopsis thaliana* and further verified by differential expression and RT-qPCR analysis. *VrFATB*, *VrGSO1*, *VrLACS2*, and *VrPAT14* around QEIs were homologous to known seed development genes in *A. thaliana*. Hong et al. measured two epicotyl length traits in 951 soybean accessions over two years and used 3VmrMLM to perform association analysis between phenotypes and 1,639,846 SNPs, identifying 180 QTNs and QEIs. Based on transcript abundance, GO enrichment, and haplotype analysis, 10 candidate genes were predicted to be involved in the process of seed germination and seedling development, and it was found that *Glyma.04G122400* and *Glyma.18G183600* may affect epicotyl length elongation. Han et al. measured the flowering time (FT) of 490 *Brassica napus* accessions in eight environments and used 3VmrMLM to perform association analysis between the phenotypes and 11,700,689 SNPs, identifying 19 stable QTNs and 32 QEIs for FT and 10 QTNs for FT-related climatic indices. A total of 12 and 14 differentially expressed genes were found to be candidate genes for stable QTNs and QEIs, respectively, while five DEGs were found to be candidate genes for the indices. *BnaFLCs*, *BnaFTs*, *BnaA02.VIN3*, and *BnaC09.PRR7* were further validated.

With the improvement in people’s living standards, crop quality traits are becoming increasingly important. Yu et al. measured four seed tocopherol content traits of 175 soybean accessions in three environments, used six methods to perform association analysis between the phenotypes and 23,149 SNPs, identifying 101 QTNs in single-environment analysis and 57 QTNs and 13 QEIs in multi-environment analysis. A total of 11 candidate genes residing in eight novel QTLs were confirmed using haplotype, RNA-Seq, and RT-qPCR analysis. Zheng et al. evaluated three cooking quality traits in 345 rice accessions over two years and used seven multi-locus methods to perform association analysis between phenotypes and 193,582 SNPs, identifying 144 QTNs and 21 QEIs. Based on analyses of mutation type, gene ontology classification, haplotype, and expression pattern, *OsSSIIIb*, *OsMT2b*, *wx*, *OsSSIIa*, and *OsSSIIIa*, which are related to starch synthesis and endosperm development, were found to influence grain expansion after cooking. Azam et al. measured the seed isoflavone accumulation of 1551 soybean accessions in five environments, used cMLM to perform association analysis between the phenotypes and 6,149,599 SNPs, and revealed that the allelic variation of *Glyma.11G108100* significantly influenced isoflavone accumulation.

Resistance, a key trait affecting crop yield, is the focus of two articles in this Research Topic. Kou et al. measured the pre-harvest sprouting of 629 Chinese wheat varieties in two environments, and they used the mrMLM and IIIVmrMLM software to perform association analysis between the phenotypes and 314,548 SNPs, identifying 22 stable QTNs for PHS resistance, such as AX-95124645 (r^2^ ≥ 36%). Importantly, all white-grained varieties with the QSS.TAF9-3DTT haplotype showed resistance to spike sprouting. Around this locus, *TraesCS3D01G466100* and *TraesCS3D01G468500* were differentially expressed and found by GO annotation to be related to pre-harvest sprouting resistance. He et al. evaluated Pasmo resistance in 445 flax accessions over 5 years and used four methods to perform association analysis between phenotypes and 246,035 SNPs, identifying 132 tag QTNs and 50 QEIs. A total of 37 and 9 resistance gene analogs were considered potential candidates for QTNs and QEIs, respectively.

In addition, Wu et al. evaluated eight traits of 226 sunflower inbred lines under control and drought stress conditions and used three methods to perform association analysis between these phenotypes and 94,162 SNPs. Among the 118 genes around 80 QTNs, 14 candidate genes were validated by RNA-seq and RT-qPCR analysis, and *LOC110885273*, *LOC110872899*, *LOC110891369*, and *LOC110920644* were found to be abscisic acid-related protein kinases and transcription factors.

## 3 Future perspectives

To effectively identify QEIs across diverse environments and QQIs across numerous markers, it is imperative to devise new algorithms tailored to sample size, computational speed, and minimal memory requirements to meet the needs of human large data analysis. As the field advances, the genetic model for quantitative traits may transition from the classic Fisher genetic model to a more comprehensive framework through the integration of artificial intelligence. We anticipate that our compressed variance component mixed model will emerge as a pivotal tool in the genetic analysis of complex traits and multi-omics data in the future.

## Author contributions

Y-MZ: Writing – original draft, Writing – review & editing. ZJ: Writing – review & editing. S-QX: Writing – review & editing. JW: Writing – review & editing. SW: Writing – review & editing. Y-WZ: Writing – review & editing.
